# CD4^+^FOXP3^+^ Regulatory T Cells Exhibit Impaired Ability to Suppress Effector T Cell Proliferation in Patients with Turner Syndrome

**DOI:** 10.1371/journal.pone.0144549

**Published:** 2015-12-28

**Authors:** Young Ah Lee, Hang-Rae Kim, Jeong Seon Lee, Hae Woon Jung, Hwa Young Kim, Gyung Min Lee, Jieun Lee, Ji Hyun Sim, Sae Jin Oh, Doo Hyun Chung, Choong Ho Shin, Sei Won Yang

**Affiliations:** 1 Department of Pediatrics, Seoul National University Children’s Hospital, Seoul National University College of Medicine, 103 Daehak-ro, Jongno-gu, Seoul, Republic of Korea; 2 Department of Anatomy, Seoul National University College of Medicine, 103 Daehak-ro, Jongno-gu, Seoul, Republic of Korea; 3 Department of Biomedical Sciences, Seoul National University College of Medicine, 103 Daehak-ro, Jongno-gu, Seoul, Republic of Korea; 4 Department of Pathology, Seoul National University Hospital, Seoul National University College of Medicine, 103 Daehak-ro, Jongno-gu, Seoul, Republic of Korea; 5 Ischemic/Hypoxia Institute, Seoul National University College of Medicine, 103 Daehak-ro, Jongno-gu, Seoul, Republic of Korea; University of Lisbon, PORTUGAL

## Abstract

**Objective:**

We investigated whether the frequency, phenotype, and suppressive function of CD4^+^FOXP3^+^ regulatory T cells (Tregs) are altered in young TS patients with the 45,X karyotype compared to age-matched controls.

**Design and Methods:**

Peripheral blood mononuclear cells from young TS patients (*n* = 24, 17.4–35.9 years) and healthy controls (*n* = 16) were stained with various Treg markers to characterize their phenotypes. Based on the presence of thyroid autoimmunity, patients were categorized into TS (–) (*n* = 7) and TS (+) (*n* = 17). Tregs sorted for CD4^+^CD25^bright^ were co-cultured with autologous CD4^+^CD25^−^ target cells in the presence of anti-CD3 and -CD28 antibodies to assess their suppressive function.

**Results:**

Despite a lower frequency of CD4^+^ T cells in the TS (-) and TS (+) patients (mean 30.8% and 31.7%, vs. 41.2%; *P* = 0.003 and *P* < 0.001, respectively), both groups exhibited a higher frequency of FOXP3^+^ Tregs among CD4^+^ T cells compared with controls (means 1.99% and 2.05%, vs. 1.33%; *P* = 0.029 and *P* = 0.004, respectively). There were no differences in the expression of CTLA-4 and the frequency of Tregs expressing CXCR3^+^, and CCR4^+^CCR6^+^ among the three groups. However, the ability of Tregs to suppress the *in vitro* proliferation of autologous CD4^+^CD25^−^ T cells was significantly impaired in the TS (–) and TS (+) patients compared to controls (*P* = 0.003 and *P* = 0.041). Meanwhile, both the TS (–) and TS (+) groups had lower frequencies of naïve cells (*P* = 0.001 for both) but higher frequencies of effector memory cells (*P* = 0.004 and *P* = 0.002) than did the healthy control group.

**Conclusions:**

The Tregs of the TS patients could not efficiently suppress the proliferation of autologous effector T cells, despite their increased frequency in peripheral CD4^+^ T cells.

## Introduction

Turner syndrome (TS) phenotypes include short stature, characteristic skeletal features, sexual infantilism, premature ovarian failure, congenital heart and kidney anomalies, obesity, insulin resistance, hearing loss, and cognitive deficits [1]. Moreover, patients with TS are at high risk of autoimmune diseases [2], although the reason for this remains unclear.

Several factors may account for the female predominance of autoimmune disease, including estrogen and/or X chromosome inactivation. Approximately 15% of X-linked genes escape inactivation, suggesting that there is a remarkable degree of expression heterogeneity among females. [3] A higher prevalence of autoimmune thyroid disease (AITD), inflammatory bowel disease, and other autoimmune diseases in TS patients compared with not only healthy females but also those with premature ovarian insufficiency [4], suggests that TS phenotypes might be attributable to the altered expression of X-linked genes [1].

Among the genes located on the X chromosome, *FOXP3* encodes a transcription factor that is critical for the function of regulatory T cells (Tregs) and plays a key role in establishing immune homeostasis [5]. *FOXP3* mutations cause fatal autoimmune lymphoproliferative diseases in humans (immunodysregulation polyendocrinopathy enteropathy X-linked syndrome) and mice (scurfy mice) [6]. Interestingly, thyroid autoimmunity in TS has been mapped to a critical region in Xp11.2–p22.1, the chromosomal region containing the *FOXP3* gene [7]. Therefore, changes in the expression and function of FOXP3 might be involved in the susceptibility of TS patients to autoimmunity.

To date, few studies have investigated whether the frequency and/or suppressive function of Tregs is altered in patients with TS [8]. The only previous study to compare the suppressive function of Tregs in patients with TS and controls found no difference between the groups [8]. A recent study found a higher frequency of Tregs in patients with TS than in healthy controls [9]. However, previous studies were limited by the inclusion of heterogeneous TS patients with different karyotypes, a variety of patient ages, and the presence of various autoimmune diseases [8,9].

In the present study, we investigated whether the frequency, phenotype, and regulatory function of CD4^+^FOXP3^+^ Tregs were altered in TS patients compared with age-matched controls. After excluding individuals with all autoimmune diseases except AITD, only young TS patients with the 45,X karyotype and age-matched controls were included.

## Materials and Methods

### Subjects

The Seoul National University Hospital Ethics Committee (H-1108-054-373) approved this study. Written informed consent was obtained from all 40 participants (24 patients with TS and 16 controls). Informed consent was also written by the parents of patients under 18 years enrolled in this study. The diagnosis of TS was confirmed by chromosome analysis, and only young patients with TS (17.4–35.7 years of age) with the 45,X karyotype were included with age-matched healthy controls (HC). All patients with TS had received previous growth hormone therapy, reached final adult height, and experienced regular menstruation with cyclic estrogen and progesterone replacement therapy. None of the HC received estrogen-based contraceptives.

With the exception of AITD, patients with TS who had diseases that affected the immune system, including diabetes, inflammatory bowel disease, vitiligo, alopecia, and asthma, or who were taking immunosuppressive drugs, were excluded. Because of the low prevalence of type 1 diabetes and celiac disease in Korean individuals compared with Caucasians [10], we found no cases of celiac disease or type 1 diabetes in our patient group. However, Hashimoto’s thyroiditis (HT) and thyroid dysfunction, the most common autoimmune conditions among Koreans, were frequently observed in our patients with TS, consistent with previous reports [2,11,12]. Although previous studies investigating the frequency of Tregs in autoimmune disease have reported mixed findings, most patients with autoimmunity appear to have a sufficient number of Tregs in their peripheral blood [13]. To consider the effect of thyroid autoimmunity per se on the frequencies and functions of Tregs [14,15], patients with TS were categorized into TS (–) (*n* = 7) and TS (+) (*n* = 17) based on the presence of thyroid autoimmunity.

The patients with TS underwent regular follow-up evaluations of serum thyroid function and thyroid autoantibodies (Abs) every 6 to 12 months. Serum concentrations of free thyroxine (FT4) and thyroid-stimulating hormone (TSH) were measured using immunoradiometric kits (RIAKEY; Shin Jin Medics, Seoul, Republic of Korea). Serum levels of triiodothyronine (T3), anti-thyroglobulin (Tg) Abs, and anti-thyroid peroxidase (TPO) Abs were measured using radioimmunoassay kits (Brahms DYNOTest; Diagnostica GmbH, Berlin, Germany). The normal ranges of serum FT4, TSH, and T3 are 0.70–1.80 ng/dL (9.01–23.2 nmol/L), 0.4–4.1 mIU/L, and 87–184 ng/dL (1336–2826 pmol/L), respectively. Thyroid autoimmunity was documented by the presence of anti-Tg Abs and/or anti-TPO Abs during the follow-up period [12].

### Flow cytometry and cell sorting

Human peripheral blood was drawn (60 mL of whole blood), and peripheral blood mononuclear cells (PBMCs) were isolated using Ficoll-Hypaque (Sigma-Aldrich, St. Louis, MO, USA) density gradient centrifugation. First, PBMCs were stained with Abs against CD4 and CD25 surface antigens, including CXCR3, CD45RA, CCR4 (R&D Systems), CCR6, and glucocorticoid-induced tumor necrosis factor receptor (GITR; BioLegend, San Diego, CA, USA). Then they were washed, permeabilized with permeabilizing buffer, and stained intracellularly with anti-FOXP3 Abs (Miltenyi Biotec, Auburn, CA, USA) or the appropriate isotype controls. Second, PBMCs were stained with anti-CD4 Abs, followed by intracellular staining with anti-FOXP3 and -cytotoxic T lymphocyte antigen-4 (CTLA-4) Abs. Third, anti-CD4, -CD8, -CD45RA, and -CCR7 Abs were used to analyze T cell subsets. For intracellular cytokine detection CD4^+^ T cells, PBMCs were stained with Abs against surface molecules, CD3 and CD8, to gate CD3^+^CD8^–^ as the marker of CD4^+^ T cells since the expression of surface CD4 molecules is down-regulated in human CD4^+^ T cells by TCR stimulation. Then, cells were stimulated for 4 h with 50 ng/mL phorbol myristate acetate (Sigma-Aldrich) and 1.0 μg/mL ionomycin (Sigma-Aldrich) in the presence of 1.0 μg/mL GolgiStop^®^ (BD Biosciences, San Jose, CA, USA). We confirmed that the frequencies of CD3^+^CD8^–^ T cells were almost the same as those of CD3^+^CD4^+^ T cells. Next, intracellular staining was performed with anti-IFN-γ, -TNF-α, -IL-4, and -IL-17 Abs, or appropriate isotype controls. In separate experiments, stimulated PBMCs were stained with Abs against IFN-γ, IL-17, and FOXP3, or appropriate isotype controls. All reagents and Abs were purchased from BD Biosciences unless otherwise specified.

All stained cells were analyzed by flow cytometry on an LSR^®^ flow cytometer (BD Biosciences) equipped with five lasers. For cell sorting into CD4^+^CD25^bright^ (Tregs) and CD4^+^CD25^–^ T cells (target cells), a FACSAria^®^ (BD Biosciences) was used. The data were analyzed using FlowJo^®^ software (TreeStar, Ashland, OR, USA).

### Inhibition assays

CD4^+^CD25^–^ T cells were labeled with carboxyfluorescein diacetate succinimidyl ester (CFSE; Molecular Probes, Eugene, OR, USA). Most CD4^+^CD25^bright^ T cells in the TS and HC groups were FOXP3-positive (S1 Fig, R1–R4). Tregs (CD4^+^CD25^bright^) were co-cultured with CFSE-labeled CD4^+^CD25^–^ target cells (3 X 10^4^ cells/well) at Treg:target cell ratios of 0:1, 0.1:1, 0.25:1, 0.5:1, and 1:1 for 6 days (S2 Fig) in the presence of anti-CD3 and -CD28 Ab-coated beads (cell to beads ratio 80:1) (Life Technologies, Grand Island, NY, USA) as previously described [16]. Stained cells were analyzed on an LSR^®^ flow cytometer and then using FlowJo^®^ software. The inhibitory index (%) of cell proliferation (or cytokine production) was calculated for each sample as follows: (proliferation [or cytokine production] of target cells alone–proliferation [or cytokine production] of target cells in the presence of Tregs) / (proliferation [or cytokine production] of target cells alone) × 100.

### Statistical analysis

All statistical analyses were performed using SPSS 19.0 (IBM SPSS Statistics, New York, NY, USA). The normality of variable distribution was tested, and variables exhibiting skewed distributions were log-transformed prior to analysis. The significance of each difference in the means of continuous variables exhibiting homogenous variance among the three groups was tested via an analysis of variance (ANOVA) followed by Bonferroni *post-hoc* subgroup analysis. Generalized estimating equations (GEEs) with normal distributions, featuring identity linkages, were used to compare the effects of Tregs on HC and TS patients in terms of suppressing the proliferation of and cytokine production by target cells. We constructed an exchangeable correlation structure to obtain within-group correlations. GEE-adjusted within-subject correlations were calculated using repeated-measures outcomes at 0.1:1, 0.25:1, 0.5:1, and 1:1 ratios of Tregs to target cells. All data are expressed as means with standard deviations. A *P*-value < 0.05 indicated a statistically significant difference.

## Results

### Participant characteristics

No significant differences in age and BMI were found among the three groups, except that the TS (−) and TS (+) patients were shorter than the HC (*P* = 0.001 for both, Table 1). Six TS patients had congenital anomalies and 3 of them received cardiac surgery. One TS (+) patient was on levothyroxine treatment (Table 1).

**Table 1:** Clinical characteristics of participants.

	HC	TS (−)	TS (+)
*n*	16	7	17
Age (years)	27.3 (4.9)	24.9 (4.3)	25.9 (6.5)
Height (cm)	160.6 (5.0)	149.5 (5.6) ^a^	152.3 (7.1) ^a^
Weight (kg)	54.5 (9.1)	48.3 (10.8)	54.4 (12.3)
BMI	21.1 (3.6)	21.5 (3.8)	23.2 (3.5)
Obesity (*n*)	2	1	2
Levothyroxine (*n*)	0	0	1
Congenital heart anomaly (*n*)	0	2	4
Congenital heart anomalies		BAV (1), AS (1)	BAV (1), AS & BAV (1), CoA & BAV (1), PDA (1)
Cardiac surgery (*n*)	0	1	2

Data are expressed as means (standard deviations).

^a^ *P* = 0.001 vs. the HC group

Abbreviations: HC, Healthy control subjects; TS (−), Turner syndrome patients without thyroid autoimmunity; TS (+), Turner syndrome patients with thyroid autoimmunity; BAV, bicuspid aortic valve; AS, aortic stenosis; CoA, coarctation of aorta; PDA, persistent ductus arteriosus.

### Higher frequency of FOXP3^+^ Tregs among CD4^+^ cells with the activated and non-suppressive phenotype in TS patients

**Fig 1:**
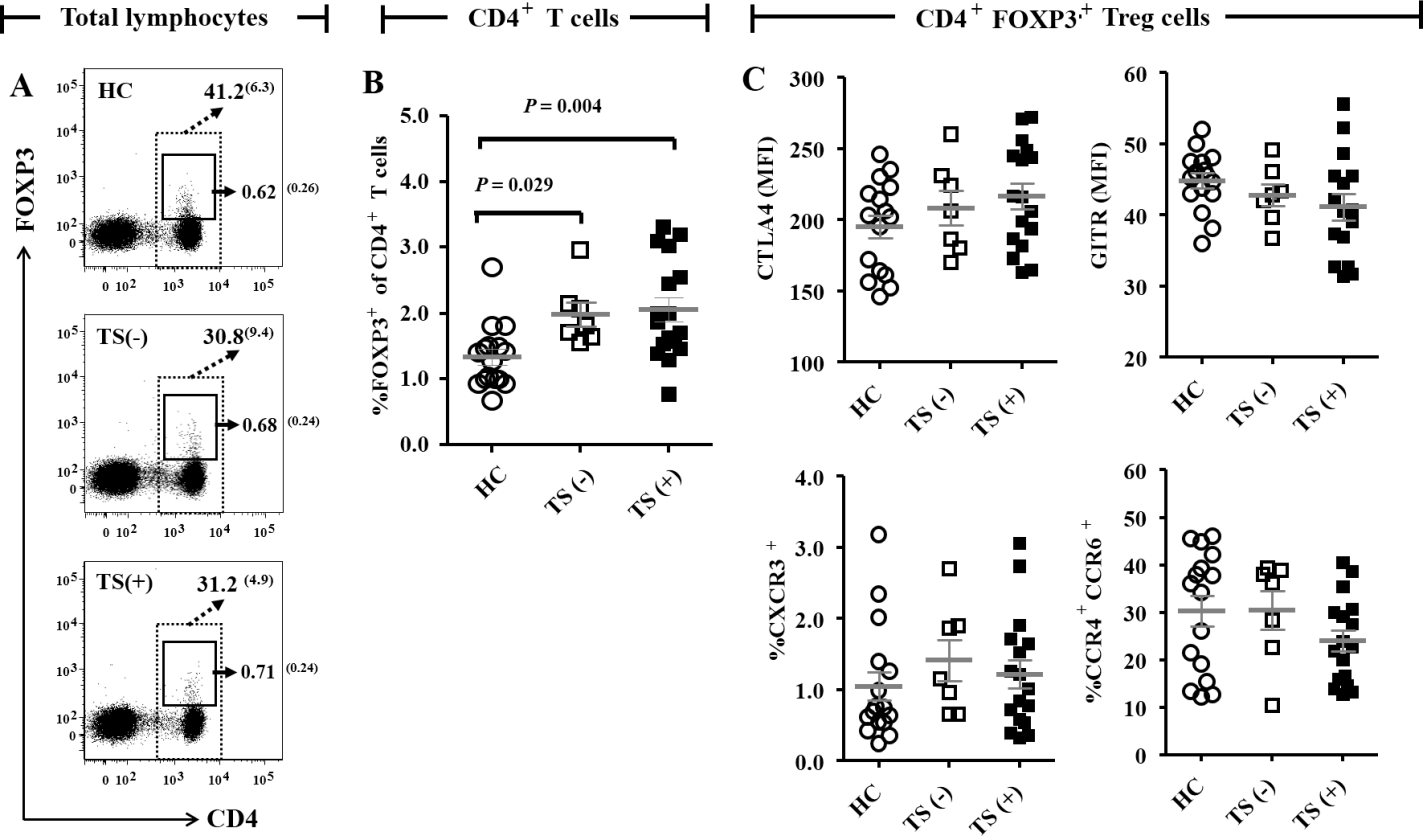
Comparison of frequency and phenotypes of CD4^+^FOXP3^+^ Tregs among the HC, TS (–) and TS (+) groups. (A) Both the TS (–) and TS (+) groups had lower frequencies of CD4^+^ T cells than the HC group as shown by the dotted boxes. The frequencies of CD4^+^FOXP3^+^ Tregs (among total lymphocytes) did not significantly differ among the three groups as shown by the lined boxes. Numbers within the plots indicate the mean (standard deviations) percentage of each subset. (B) The TS (–) and TS (+) groups exhibited higher percentages of FOXP3^+^ among their CD4^+^ T cells than did the HC group. (C) There were no significant differences among the HC, TS (–) and TS (+) groups in the expression of CTLA-4 and GITR in CD4^+^FOXP3^+^ Tregs. The percentages of CD4^+^FOXP3^+^ Tregs expressing CXCR3, or both CCR4 and CCR6 were also comparable among the three groups. Grey bars and error bars indicate means and standard deviations, respectively. Values from the three groups were compared by ANOVA, and the between-group values were compared via Bonferroni *post-hoc* analysis. Abbreviations: HC, healthy control subjects; TS (−), Turner syndrome patients without thyroid autoimmunity; TS (+), Turner syndrome patients with thyroid autoimmunity; CTLA4, cytotoxic T lymphocyte antigen-4; MFI, mean fluorescence intensity; GITR, glucocorticoid-induced tumor necrosis factor receptor family related.

We measured the frequency and phenotypic changes in T cell subsets, including CD4^+^FOXP3^+^ Tregs, using flow cytometry. We observed significant differences in the frequencies of T cell subsets, including CD4^+^ T cells (*P* < 0.001), and the CD4/CD8 ratio (*P* = 0.002) among the three groups (Fig 1A, S1 Table). Both the TS (–) and TS (+) groups had lower frequencies of CD4^+^ T cells (mean values 30.8% and 31.7%, respectively, vs. 41.2%; *P* = 0.003 and *P* < 0.001, respectively, Fig 1A). In contrast, there were no significant differences in the frequency of CD8^+^ T cells among the three groups. The CD4/CD8 ratio was significantly lower in TS (+) than the HC group (mean values 1.10, vs. 1.71; *P* = 0.002, S1 Table). The percentages of CD4^+^FOXP3^+^ Tregs among total lymphocytes did not significantly differ among the three groups (Fig 1A).

Significant differences in the percentages of FOXP3^+^ (among CD4^+^ T cells) were evident among the three groups (*P* = 0.003 for both comparisons between HC and both TS groups, Fig 1B). The TS (–) and TS (+) groups exhibited higher percentages of FOXP3^+^ cells (means 1.99% and 2.05%, respectively, vs. 1.33%; *P* = 0.029 and *P* = 0.004, respectively) among CD4^+^ T cells than did the HC group (Fig 1B). This suggests that the Treg frequency among CD4^+^ T cells increased in patients with TS regardless of the development of thyroid autoimmunity.

**Fig 2:**
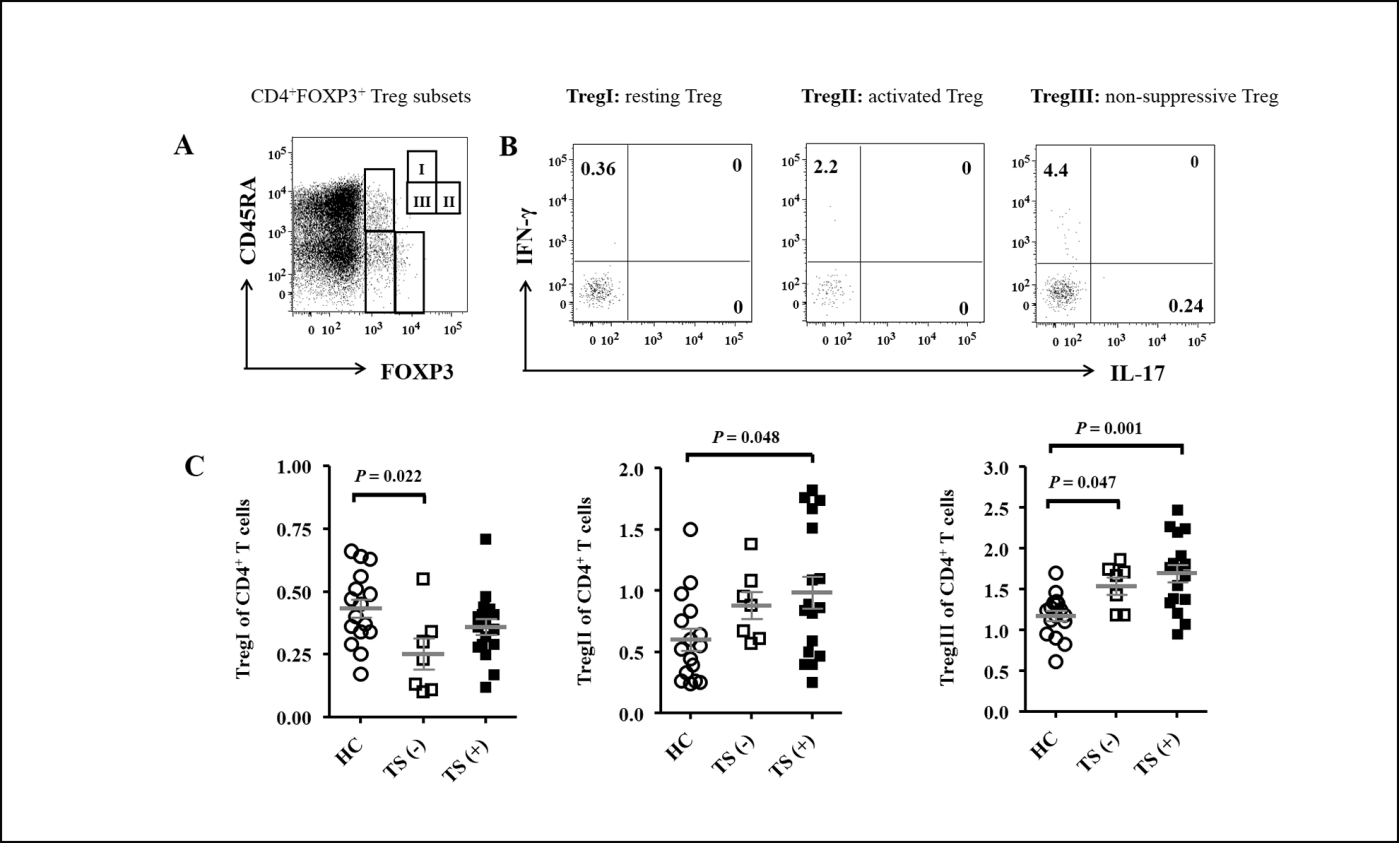
Comparison of the frequency of CD4^+^FOXP3^+^ Treg subsets according to CD45RA expression between the TS and HC groups. (A) PBMCs were stained with Abs against CD4, CD45RA, and FOXP3, and then analyzed by flow cytometry. CD4^+^FOXP3^+^ Tregs were categorized into three distinct subsets: CD45RA^+^FOXP3^lo^ resting Treg (TregI), CD45RA^–^FOXP3^hi^ activated Treg (TregII), and CD45RA^–^FOXP3^lo^ non-suppressive Treg (TregIII) cells. (B) Then cytokine production in the Treg subsets was measured by stimulating PBMCs with PMA and ionomycin in the presence of GolgiStop®, and by intracellular staining with Abs against IFN-γ and IL-17. Numbers on the dot plot indicate the frequency of cytokine-producing cells. (C) While the frequency of TregI cells was lower in the TS (–) than in the HC group, the frequency of TregII cells was higher in the TS (+) than in the HC group. The frequency of TregIII cells was higher in the TS (–) and TS (+) than in the HC group. Grey bars and error bars indicate means and standard deviations, respectively. Values from the three groups were compared by ANOVA, and the between-group values were compared via Bonferroni *post-hoc* analysis. Abbreviations: Tregs, regulatory T cells; HC, healthy control subjects; TS (−), Turner syndrome patients without thyroid autoimmunity; TS (+), Turner syndrome patients with thyroid autoimmunity.

Next, we further characterized CD4^+^FOXP3^+^ Tregs based on the expression of various markers that might represent their function to determine whether there were changes in the frequency of Treg subsets and in their phenotypfes. CD4^+^FOXP3^+^ Tregs were divided into three distinct Treg subsets (Fig 2A) based on the expression of CD45RA and FOXP3, as described previously [17]: CD45RA^+^FOXP3^lo^ resting Treg (TregI), CD45RA^–^FOXP3^hi^ activated Treg (TregII), and CD45RA^–^FOXP3^lo^ non-suppressive Treg (TregIII) cells. Consistent with a previous publication [17], the TregIII cells produced more IFN-γ and IL-17 than did the TregI or TregII cells (Fig 2B). The cytokine profiles of the different Treg subsets did not differ among the HC, TS (–) and TS (+) groups (data not shown). However, the frequencies of TregI (*P* = 0.024), TregII (*P* = 0.036), and TregIII cells (*P* = 0.001) were significantly different among the three groups. While the frequency of TregI cells was lower in the TS (–) than in the HC group (mean values 0.25% vs. 0.43%; *P* = 0.022), the frequency of TregII cells was higher in the TS (+) than in the HC group (mean values 0.98% vs. 0.60%; *P* = 0.048, Fig 2C). The frequency of TregIII cells was higher in both the TS (–) and TS (+) groups than in the HC group (mean values 1.54% and 1.69%, respectively, vs. 1.17%; *P* = 0.047 and *P* < 0.001, respectively, Fig 2C). This indicates that the heterogeneities of Tregs may affect their regulatory function.

Then, we measured the expression of CTLA-4, GITR, and several chemokine receptors in CD4^+^FOXP3^+^ Tregs to determine the function of the Tregs. We found no significant differences among the HC, TS (-) and TS (+) groups in the expression of CTLA-4 and GITR in CD4^+^FOXP3^+^ Tregs. The percentages of CD4^+^FOXP3^+^ Tregs expressing CXCR3, or both CCR4 and CCR6 were also comparable among the three groups (Fig 1C).

### The ability of CD4^+^CD25^bright^ Tregs to suppress autologous CD4^+^CD25^-^ T cell proliferation in TS patients is defective

Although we identified a higher frequency of CD4^+^FOXP3^+^ Tregs in TS patients, their immunosuppressive function might be different in TS patients and controls. We evaluated the suppressive function of CD4^+^CD25^bright^ T cells, as Tregs, by measuring the proliferation and cytokine production of autologous CD4^+^CD25^−^ T cells, as target cells, in vitro.

**Fig 3:**
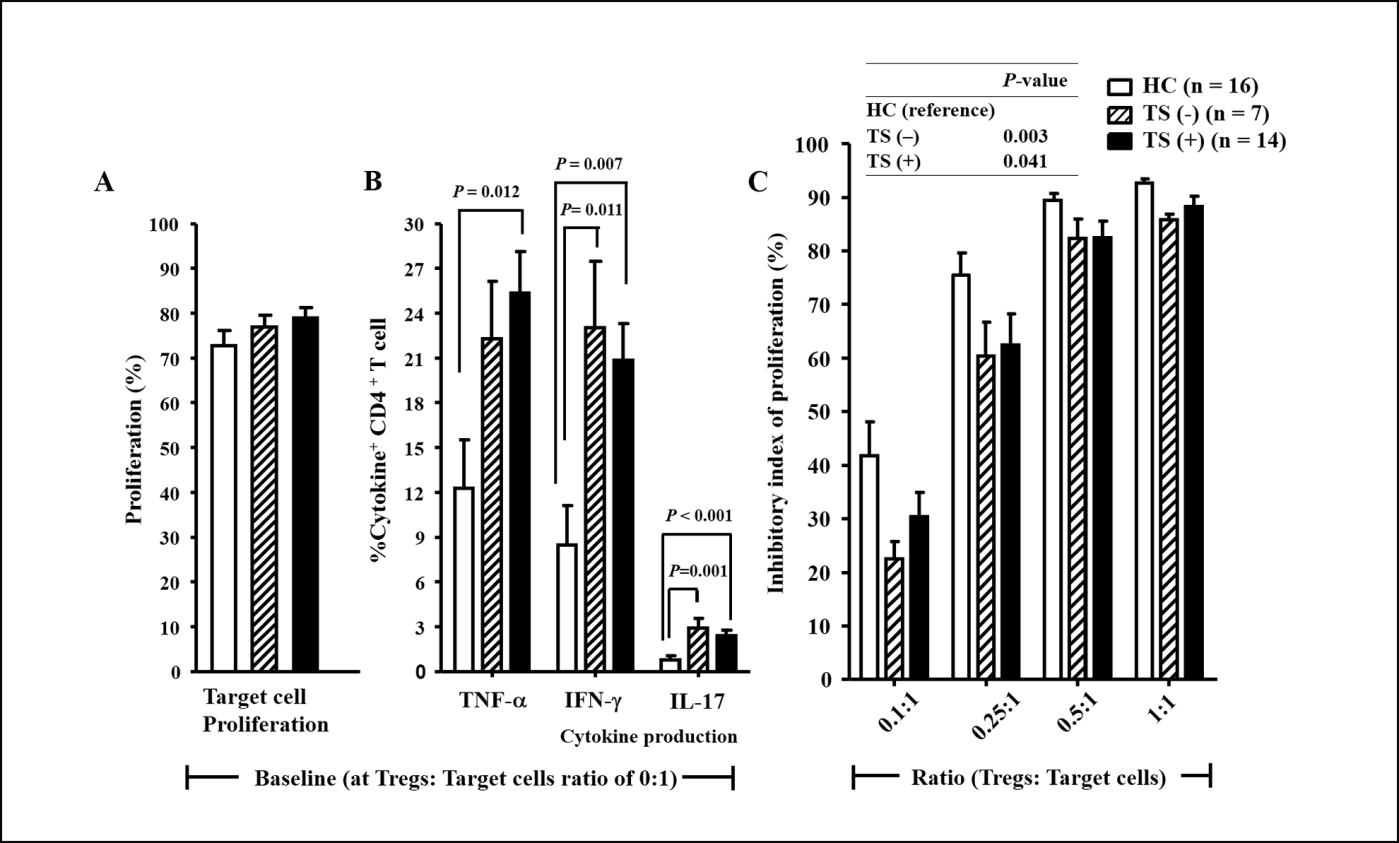
Baseline target cell proliferation and cytokine production and inhibitory index of proliferation among the HC, TS (–) and TS (+) groups. Whereas baseline target cell proliferation (at Treg: target cell ratio of 0:1) was similar among the three groups (A), CD4+CD25– target cells from TS (–) and TS (+) patients produced higher levels of TNF-α, IFN-γ, and IL-17 compared to cells from the HC group (B). The percentage of the inhibitory index of cell proliferation was calculated as described in Methods. The inhibitory index of proliferation indicating the suppressive function of Tregs was significantly lower in the TS (–) and TS (+) than the HC group (C). Bars and error bars indicate means and standard deviations, respectively. Values (B) from the three groups were compared by ANOVA, and the between-group values were compared via Bonferroni *post-hoc* analysis. P-values (C) were calculated using generalized estimating equations. Abbreviations: HC, healthy control subjects; TS (−), Turner syndrome patients without thyroid autoimmunity; TS (+), Turner syndrome patients with thyroid autoimmunity.

Whereas baseline target cell proliferation (at Treg: target cell ratio of 0:1) was similar among the three groups (Fig 3A), CD4^+^CD25^–^ target cells from TS (–) and TS (+) patients produced higher levels of TNF-α (*P* = 0.194 and *P* = 0.012, respectively), IFN-γ (*P* = 0.011 and *P* = 0.007, respectively), and IL-17 (*P* = 0.001 and *P* < 0.001, respectively) compared to cells from the HC (Fig 3B). Meanwhile, target cell proliferation and the production of TNF-α, IFN-γ, and IL-17 in target cells did not differ between the TS (–) and TS (+) patients (Fig 3A and). This suggests that effector T cells from TS patients are activated and resistant to Tregs compared to those from HC regardless of the presence of thyroid autoimmunity.

As a result of GEE-adjusted within-subject correlations using repeated-measures outcomes at 0.1:1, 0.25:1, 0.5:1, and 1:1 ratios of Tregs to target cells, the inhibitory index of proliferation indicating the suppressive function of Tregs was significantly lower in the TS (–) and TS (+) than the HC group (*P* = 0.003 and *P* = 0.041, respectively, Fig 3C). However, target cell proliferation was suppressed at similar levels between the TS (–) and TS (+) patients (Fig 3C). The production of TNF-α, IFN-γ, and IL-17 in target cells were suppressed at similar levels among the three groups (data not shown). Taken together, the suppressive function of Tregs was significantly impaired in patients with TS regardless of thyroid autoimmunity.

### Higher frequency of effector memory CD4^+^ T cells in TS patients

**Fig 4:**
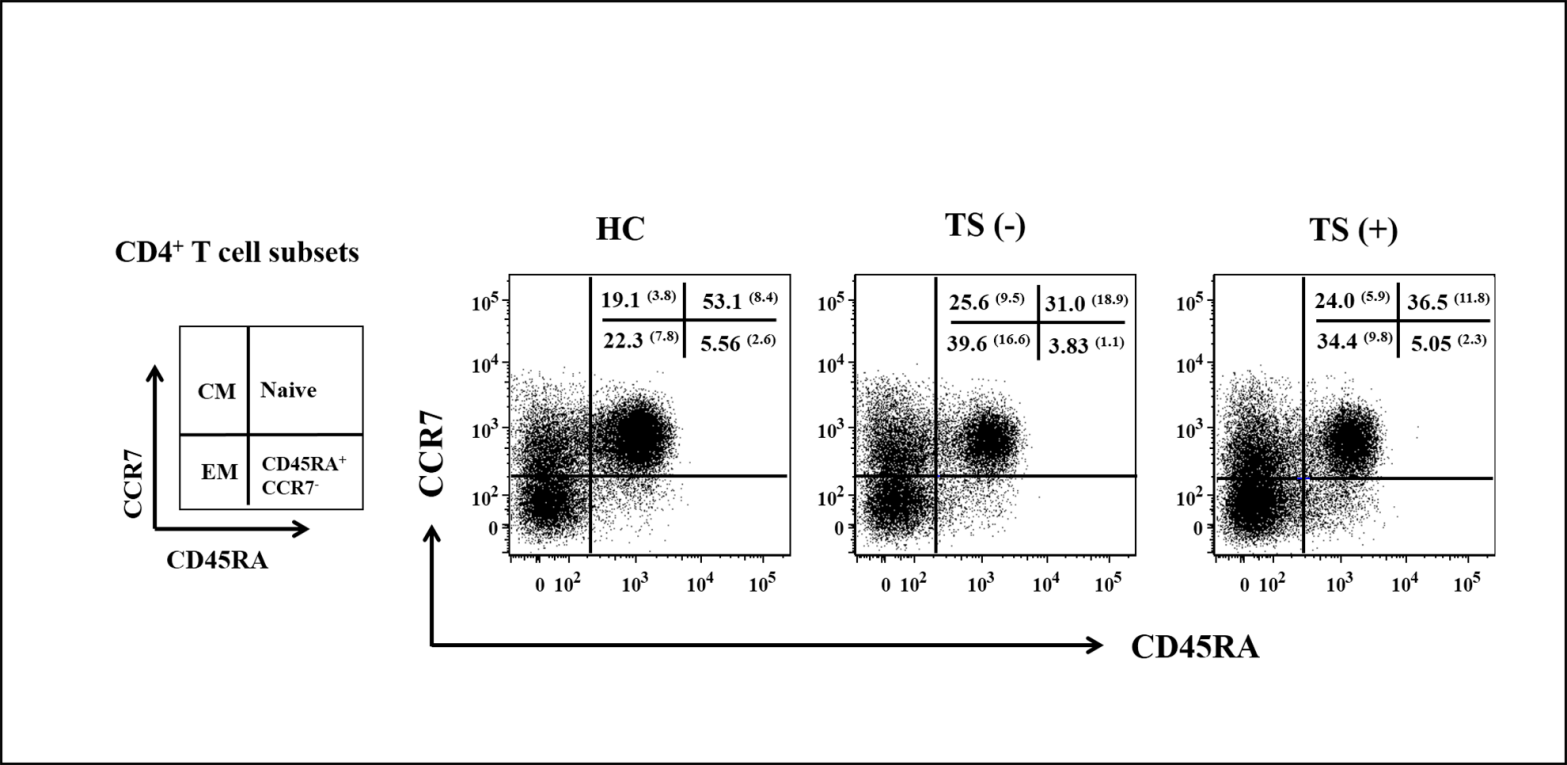
Comparison of CD4^+^ T cell subsets among the HC, TS (−), and TS (+) groups. Both the TS (–) and TS (+) groups had lower frequencies of naïve cells (*P* = 0.001 for both) but higher frequencies of EM cells than did the HC group. Numbers within the plots indicate the mean (standard deviations) percentage of each subset. Values from the three groups [HC, TS (-), and TS (+)] were compared by ANOVA, and the between-group values were compared via Bonferroni *post-hoc* analysis. Abbreviations: HC, healthy control subjects; TS (−), Turner syndrome patients without thyroid autoimmunity; TS (+), Turner syndrome patients with thyroid autoimmunity.

Based on the results shown above, we investigated whether effector T cell subsets in TS patients have activated phenotypes, which would presumably lead to changes in the suppressive function of CD4^+^FOXP3^+^ Tregs. Human T cells can be classified as naïve (CD45RA^+^CCR7^+^), central memory (CM, CD45RA^–^CCR7^+^), and effector memory (EM, CD45RA^–^CCR7^–^) cells based on the expression of CCR7 and CD45RA (Fig 4) [18]. The observed differences in the frequencies of naïve, CM, and EM cells among CD4^+^ T cells were significant among comparisons of the three groups (*P* < 0.001, *P* = 0.029, and *P* = 0.001, respectively). As expected, both the TS (–) and TS (+) groups had lower frequencies of naïve cells (means 31.0% and 36.5%, respectively, vs. 53.1%; *P* = 0.001 for both) but higher frequencies of EM cells (means 39.6% and 34.4%, respectively, vs. 22.3%; *P* = 0.004 and *P* = 0.002, respectively) than did the HC group (Fig 4, S1 Table). This suggests that the numbers of EM cells increased in patients with TS before the development of thyroid autoimmunity. However, there was no difference in the percentage of CD4^+^ T cells expressing IFN-γ^+^, TNF-α^+^, IL-4^+^, or IL-17^+^ among the three groups (S1 Table).

## Discussion

Tregs play a crucial role in suppressing proliferation and cytokine production in T cells to regulate inflammation and/or prevent autoimmunity. Despite a lower frequency of CD4^+^ T cells, patients with TS exhibited a higher frequency of FOXP3^+^ Tregs among CD4^+^ T cells compared with controls. Moreover, patients with TS had higher frequencies of EM CD4^+^ T cells than controls, but a lower frequency of naïve CD4^+^ T cells. The frequency of CD4^+^FOXP3^+^ Tregs expressing CTLA-4, GITR^+^, CXCR3^+^, and CCR4^+^CCR6^+^ was comparable between the patients with TS and the controls. However, the ability to suppress the *in vitro* proliferation of autologous CD4^+^CD25^−^ T cells was impaired in the Tregs of patients with TS compared with the controls. In contrast, the ability of Tregs to suppress cytokine production remained intact.

The frequency of FOXP3^+^ Tregs among CD4^+^ T cells was significantly higher in the patients with TS than in the HC regardless of the presence of thyroid autoimmunity (Fig 1B). Meanwhile, the expression of CTLA-4 in Tregs, which sends inhibitory signals to T cells via a cell-cell contact-dependent mechanism [19], did not differ between the TS and HC groups. The expression of chemokine receptors, which play roles in the recruitment of Tregs to inflamed nonlymphoid tissues [19], did not differ between the TS and HC groups. Interestingly, the patients with TS had a higher frequency of EM CD4^+^ T cells compared with the controls (Fig 4). EM CD4^+^ T cells differentiate upon antigenic stimulation, and/or become highly activated to display immediate effector responses, and then migrate to inflamed tissue [18]. Therefore, when CD4^+^CD25^–^ T cells were stimulated with anti-CD3 and -CD28 Abs for 6 days, the significantly higher levels of TNF-α, IFN-γ, and IL-17 production observed in cells from patients with TS compared with cells from the controls (Fig 3B) may be secondary to the increase in memory phenotype CD4^+^ T cells and the resultant increase in inflammatory cytokines. Taken together, these data suggest that the increase in FOXP3^+^ Tregs among CD4^+^ T cells in patients with TS is a compensatory response to suppress activated and inflammatory cytokine-producing effector CD4^+^ T cell responses.

Despite the higher frequency of Tregs in the peripheral CD4^+^ T cells of patients with TS, they were unable to efficiently suppress the *in vitro* proliferation of autologous effector CD4^+^ T cells (Fig 3C), whereas their ability to suppress cytokine production was preserved. One possible explanation for this is that the Tregs of patients with TS are intrinsically defective at inhibiting the proliferation of effector T cells, although the precise mechanism for this is unclear. The *in vivo* suppressive mechanisms of human Tregs are not yet fully elucidated, but might include modulation of the cytokine microenvironment, metabolic disruption of target cells, and functional changes in antigen presenting cells (APCs) [5,19]. In particular, the inhibitory CTLA-4 expressed by Tregs is known to modulate the activation of effector T cells by interacting with CD80/CD86 expressed on APCs [19]. FOXP3 regulates the levels of constitutively expressed CTLA-4 in Tregs [20]. The frequency of Tregs was not decreased and the expression of CTLA-4 comparable in patients with TS, probably ruling out FOXP3 haploinsufficiency. However, it is possible that other X-linked genetic factors contribute to the defective function of Tregs.

The higher frequency of CD45RA^–^FOXP3^lo^ TregIII present within the CD4^+^CD25^bright^ Tregs of patients with TS (Fig 2C) does not contribute to the overall suppressive ability of the total Treg population, but might interfere with detection of the suppressive function of authentic Tregs [17,21]. Although the higher frequency of CD45RA^–^FOXP3^hi^ activated Tregs (TregII) could contribute to suppression of the immune response, the TregIII subpopulation does not have a suppressive function, but instead produces inflammatory cytokines (Fig 2B) [17,21]. Furthermore, it was speculated that effector CD4^+^ T cells in patients with TS may be resistant to the suppressive effects of Tregs. The effector CD4^+^ T cells of TS patients produced significantly greater amounts of TNF-α, IFN-γ, and IL-17 in co-culture systems compared with those of controls (Fig 3B), although both groups retained a comparable ability to suppress cytokine production. The inflammatory cytokine milieu might play a role in the activation of effector T cells and/or override the ability of Tregs to suppress effector T cell proliferation.

Our study was limited by the small sample size, and the lack of organ-specific evaluations such as thyroid tissue. Moreover, we did not evaluate the absolute numbers of CD4^+^FOXP3^+^ Tregs in patients with TS and the HC. Several previous studies [9,22,23] have found no difference in leukocyte numbers and subpopulations between patients with TS and HC; thus, we believe a difference in lymphocyte number would have been unlikely in our study. However, because the percentage of CD4^+^FOXP3^+^ lymphocytes was comparable among the three groups, the absolute number of CD4^+^FOXP3^+^ Tregs would probably not be decreased in patients with TS compared with controls, as shown previously [9]. However, our study is strengthened by the strictly controlled inclusion criteria limiting the TS group to young individuals aged 17–36 years with the 45,X karyotype. Moreover, to the best of our knowledge, ours is the first study to compare effector T cell subsets and the phenotypic and functional characteristics of Tregs concurrently in patients with TS and age-matched controls.

## Conclusions

In conclusion, the Tregs of TS patients could not efficiently suppress the proliferation of autologous effector T cells compared to controls, despite their increased frequency in peripheral CD4^+^ T cells. It has been speculated that the Tregs of TS patients are intrinsically defective at inhibiting the proliferation of effector T cells, and/or that the effector T cells of TS patients are resistant to the suppressive effects of Tregs. Although *FOXP3* itself might not be a haploinsufficient gene, other X-linked genetic factors may contribute either to the defective function of Tregs or to activation of the autoimmune response. The mechanism underlying the defective ability of Tregs to suppress autologous effector T cells in TS patients should be elucidated in a future study.

## Supporting Information

S1 Fig Comparison of the ability of CD4^+^CD25^bright^ T cells from the TS and HC groups to suppress the proliferation of CD4^+^CD25^−^ T cells. PBMCs were stained with antibodies against CD4 and CD25, and then sorted into CD4^+^CD25^bright^ (R1, as Tregs) and CD4^+^CD25^−^ (R4, as target cells) T cells. Representative CD4^+^ T-cell subsets according CD25 expression resulted in four groups (CD25^bright^, CD25^medium^, CD25^dim^, and CD25^–^); most CD4^+^CD25^bright^ T cells were FOXP3^+^ Tregs.
(TIF)

S2 Fig Coculture of sorted CD4^+^CD25^bright^ Tregs with CFSE-labeled CD4^+^CD25^–^ target cells at various Tregs/target cells ratios. Proliferating target cells were identified based on CFSE staining using flow cytometry. The numbers on the histograms indicate the frequency of proliferating target cells. Representative data are shown from the HC and TS groups.
(TIF)

S1 Table Comparison of T cell subsets between the TS patients and healthy control subjects (DOC)
