# Supplementary figures and images for: CD4+FOXP3+ Regulatory T Cells Exhibit Impaired Ability to Suppress Effector T Cell Proliferation in Patients with Turner Syndrome
Source: PLoS One. 2015 Dec 28;10(12):e0144549. doi: 10.1371/journal.pone.0144549 (PMC4692523; doi:10.1371/journal.pone.0144549)

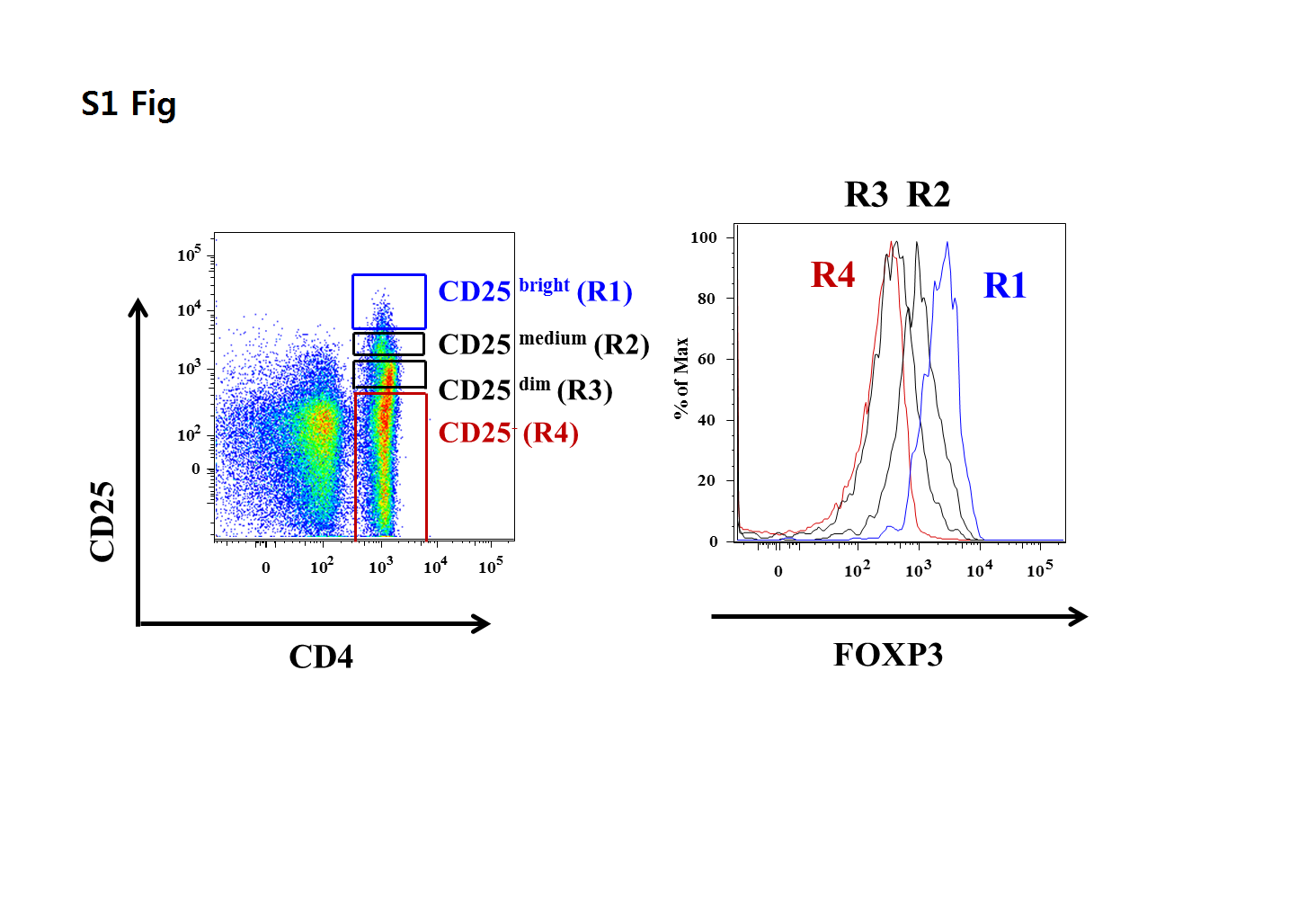

Supplement: S1 Fig — PBMCs were stained with antibodies against CD4 and CD25, and then sorted into CD4+CD25bright (R1, as Tregs) and CD4+CD25− (R4, as target cells) T cells. Representative CD4+ T-cell subsets according CD25 expression resulted in four groups (CD25bright, CD25medium, CD25dim, and CD25–); most CD4+CD25bright T cells were FOXP3+ Tregs. (TIF) [file pone.0144549.s001.tif]

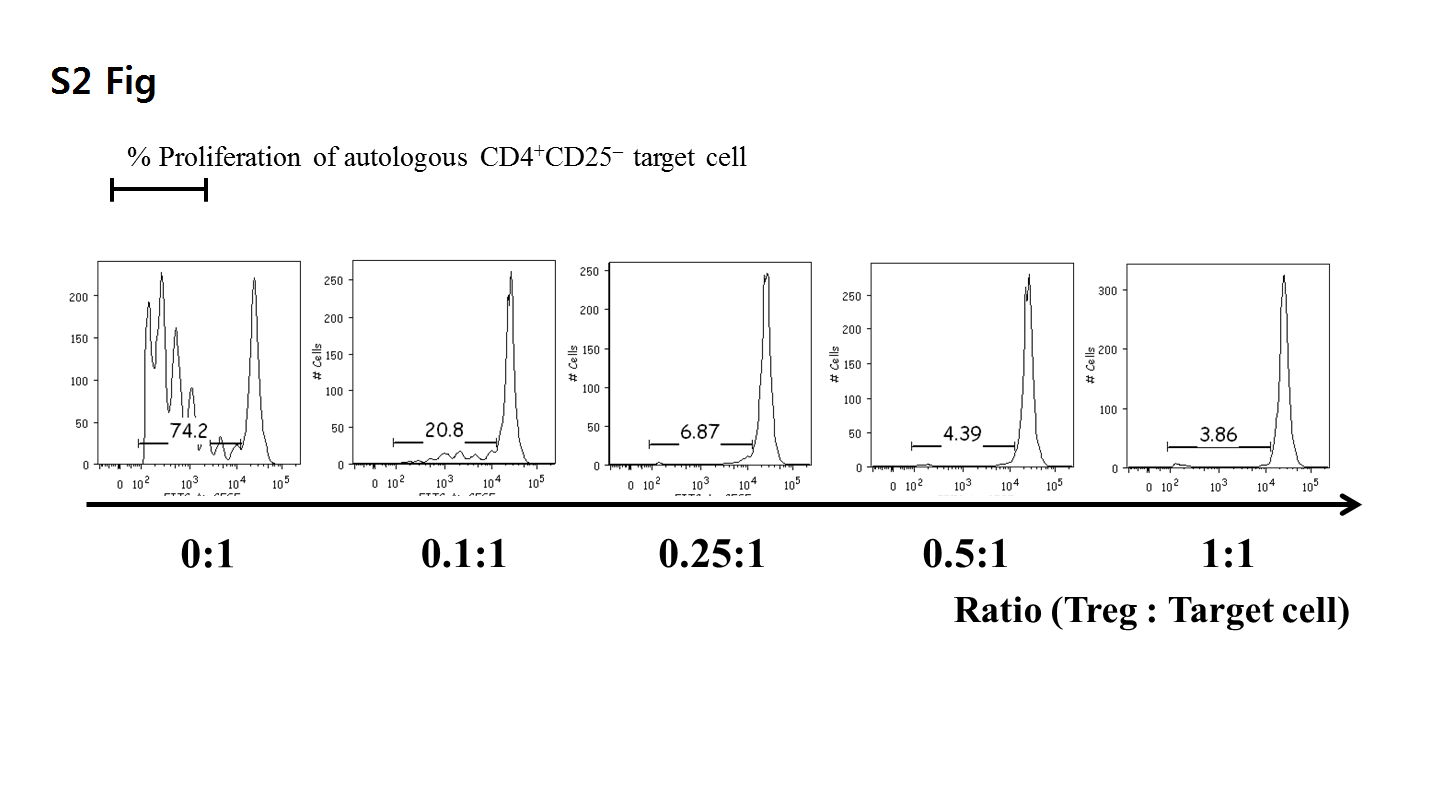

Supplement: S2 Fig — Proliferating target cells were identified based on CFSE staining using flow cytometry. The numbers on the histograms indicate the frequency of proliferating target cells. Representative data are shown from the HC and TS groups. (TIF) [file pone.0144549.s002.tif]
