# Supplementary material for: CD4+FOXP3+ Regulatory T Cells Exhibit Impaired Ability to Suppress Effector T Cell Proliferation in Patients with Turner Syndrome
Source: PLoS One. 2015 Dec 28;10(12):e0144549. doi: 10.1371/journal.pone.0144549 (PMC4692523; doi:10.1371/journal.pone.0144549)
Supplement: S1 Table — (DOC) [file pone.0144549.s003.doc]

|  |  | HC | TS (−) | TS (+) | P-value |
| --- | --- | --- | --- | --- | --- |
| *n* |  | 16 | 7 | 17 |  |
| Lymphocytes |  |  |  |  |  |
|  | %CD4^+^ T cells | 41.2 (6.3) | 30.8 (9.4) ^b^ | 31.7 (4.9) ^a^ | <0.001 |
|  | %CD8^+^ T cells | 25.4 (5.3) | 28.2 (9.1) | 30.1 (6.4) | 0.133 |
|  | CD4/CD8 ratio | 1.71 (0.5) | 1.21 (0.6) | 1.10 (0.4) ^b^ | 0.002 |
| CD4^+^ T cell subsets |  |  |  |  |  |
|  | % Naive | 53.1 (8.4) | 31.0 (18.9) ^b^ | 36.5 (11.8) ^b^ | < 0.001 |
|  | % Central memory | 19.1 (3.8) | 25.6 (9.5) | 24.0 (5.9) | 0.029 |
|  | % Effector memory | 22.3 (7.8) | 39.6 (16.6) ^b^ | 34.4 (9.8) ^b^ | 0.001 |
|  | % CD45RA^+^CCR7^–^ | 5.56 (2.6) | 3.83 (1.1) | 5.05 (2.3) | 0.303 |
| Cytokine-producing CD4^+^ T cells |  |  |  |  |  |
|  | % IFN-γ^+^ | 7.27 (4.67) | 13.8 (5.55) | 10.5 (5.80) | 0.357 |
|  | % TNF-α^+^ | 18.2 (7.1) | 20.8 (8.9) | 18.6 (5.4) | 0.694 |
|  | % IL-4^+^ | 0.32 (0.13) | 0.39 (0.29) | 0.32 (0.18) | 0.865 |
|  | % IL-17^+^ | 0.14 (0.11) | 0.17 (0.07) | 0.17 (0.09) | 0.217 |

**S1 Table.** Comparison of T cell subsets between the TS patients and healthy control subjects

The data are expressed as means (standard deviations). Variables were explored in terms of normal distribution. Certain variables (% Central memory, % Effector memory, % CD45RA^+^CCR7^–^, % IFN-γ^+^, % IL-4^+^, and % IL-17^+^) exhibited skewed distributions and were accordingly log-transformed prior to analysis. Values from the three groups [healthy control, TS (-), and TS (+)] were compared by ANOVA, and the between-group values were compared via Bonferroni *post-hoc* analysis (^a^ *P* < 0.001, ^b^ *P* < 0.01 vs. the HC group). Abbreviations: HC, healthy control subjects; TS (−), Turner syndrome patients without thyroid autoimmunity; TS (+), Turner syndrome patients with thyroid autoimmunity
